# A Generalized Laser Simulator Algorithm for Mobile Robot Path Planning with Obstacle Avoidance

**DOI:** 10.3390/s22218177

**Published:** 2022-10-25

**Authors:** Aisha Muhammad, Mohammed A. H. Ali, Sherzod Turaev, Rawad Abdulghafor, Ibrahim Haruna Shanono, Zaid Alzaid, Abdulrahman Alruban, Rana Alabdan, Ashit Kumar Dutta, Sultan Almotairi

**Affiliations:** 1 Department of Mechatronics Engineering, Faculty of Technology, Bayero University, Kano 700241, Nigeria; 2 Department of Mechanical Engineering, Faculty of Engineering, University of Malaya, Kuala Lumpur 50603, Malaysia; 3 Department of Computer Science and Software Engineering, College of Information Technology, United Arab Emirates University, Al-Ain P.O. Box 15556, United Arab Emirates; 4 Department of Computer Science, Faculty of Information and Communication Technology, International Islamic University Malaysia, Kuala Lumpur 53100, Malaysia; 5 Department of Electrical Engineering, Faculty of Technology, Bayero University, Kano 700241, Nigeria; 6 Department of Computer Science, Faculty of Computer and Information Systems, Islamic University of Medinah, Medinah 42351, Saudi Arabia; 7 Department of Information Technology, College of Computer and Information Sciences, Majmaah University, Al Majmaah 11952, Saudi Arabia; 8 Department of Information Systems, Faculty of Computer and Information Sciences College, Majmaah University, Al Majmaah 11952, Saudi Arabia; 9 Department of Computer Science and Information Systems, College of Applied Sciences Al Maarefa University, Riyadh 13713, Saudi Arabia; 10 Department of Natural and Applied Sciences, Faculty of Community College, Majmaah University, Majmaah 11952, Saudi Arabia

**Keywords:** path planning, wheeled mobile robot, generalized laser simulator, local path panning, global path planning, obstacle

## Abstract

This paper aims to develop a new mobile robot path planning algorithm, called generalized laser simulator (GLS), for navigating autonomously mobile robots in the presence of static and dynamic obstacles. This algorithm enables a mobile robot to identify a feasible path while finding the target and avoiding obstacles while moving in complex regions. An optimal path between the start and target point is found by forming a wave of points in all directions towards the target position considering target minimum and border maximum distance principles. The algorithm will select the minimum path from the candidate points to target while avoiding obstacles. The obstacle borders are regarded as the environment’s borders for static obstacle avoidance. However, once dynamic obstacles appear in front of the GLS waves, the system detects them as new dynamic obstacle borders. Several experiments were carried out to validate the effectiveness and practicality of the GLS algorithm, including path-planning experiments in the presence of obstacles in a complex dynamic environment. The findings indicate that the robot could successfully find the correct path while avoiding obstacles. The proposed method is compared to other popular methods in terms of speed and path length in both real and simulated environments. According to the results, the GLS algorithm outperformed the original laser simulator (LS) method in path and success rate. With application of the all-direction border scan, it outperforms the A-star (A*) and PRM algorithms and provides safer and shorter paths. Furthermore, the path planning approach was validated for local planning in simulation and real-world tests, in which the proposed method produced the best path compared to the original LS algorithm.

## 1. Introduction

One of the most significant processes in the autonomous navigation is path planning [1]. Path planning involves the determination of a possible path for a mobile robot to move from a start to a target location in a particular environment while considering optimization parameters like path distance, time and path smoothness [2,3,4]. As a result, the mobile Robot is expected to reach its destination within the shortest time.

Robots have been proven to be useful in various industries, including in areas which are inaccessible to humans. In recent years, autonomous navigation and path planning have attracted attention for a wide range of applications in which robots must operate in complex and hazardous environments [5]. A large amount of work is being performed to develop an intelligent algorithm that can be applied to navigating a mobile robot without a need for manual assistance.

Path planning is divided into two types, namely global and local path planning. In global planning, the autonomous robot requires information about the environment, the starting and end locations, and the positions of obstacles (all of which deal with an entirely known environment). In contrast, such information is not known (somewhat known and unknown environment) in local path planning [6].

There are two categories of obstacle settings, namely static and dynamic obstacles. In the former, the complete path must be determined prior of the start of robot movement; however, in the latter, replanning in real time is often required in dynamic or partially unknown environments, which takes a long time for path determination. The path-planning problem can be described briefly in the following steps: firstly, a feasible path for the robot must be determined based on defined start and goal positions in both known and unknown environmental settings. Secondly, the mobile robot should be able to avoid collisions with dynamic and static obstacles. Additionally, the mobile robot should complete the obstacle avoidance and pathfinding tasks using the shortest path and the least amount of time.

The advancement of automation and in-depth research in autonomous navigation technology has facilitated an increase in the use of autonomous robots in a wide range of industrial applications, including nuclear power plants using Grid-based rapidly random exploring trees *GB*-RRT* [7], space exploration using Ordered Upwind Method based Direction-dependent optimal path planning (OUM-BD) [8], rescue missions, mines, and war zones [9,10]. Planning algorithms are also helpful for frequent operations in static environments wherein optimality is needed (for example, industrial applications) [11,12,13,14].

To achieve the path-planning goal, many factors should be taken into consideration, such as the obstacle and map borders. Furthermore, factors such as static/dynamic obstacles and complete/partial unknown environments increase the difficulty of handling the path-planning problem. In an unknown setting, the mobile robot must execute simultaneous localization and mapping while exploring the environment, known as the simultaneous localization and mapping (SLAM) problem [5]. Although robot path planning in a known environment with static obstacles is considered an easy task, path planning in a dynamic environment is a challenging and fascinating area of robotics research.

This paper covers an implementation of the novel generalized laser simulator to address static and dynamic obstacles. It is organized as follows: Section two highlights the contribution, background, and a brief discussion on the related works reported on path planning. The formulation of the generalized laser simulator approach and obstacle avoidance process is introduced in Section 3. Section 4 shows the simulation and experimental results for local and global planning by the GLS algorithm. Finally, a conclusion is drawn and presented in Section 5.

## 2. Background and Related Works

Mobile robot path planning is a subcategory of trajectory planning. The aim is to determine an optimal path from a defined start to a goal point without considering kinematics and control inputs [15,16].

A feasible path for the robot in a predictable environment can be generated using global path planning approaches. Global planning methods are searching for an optimal path in known environments which is particularly successful static environments. Some of the most popular methods include Voronoi diagrams [17,18], visibility graphs and adaptive roadmaps [19], and virtual force fields (VFF) [20].

In contrast, local planning is built based on a dynamic approach that relies entirely on local perception instead of knowing the entire environment. Since the workspace is uncertain, local planning guides the mobile robots to detect nearby obstacles and take appropriate action. To avoid unexpected problems that may occur with dynamic obstacles, reactive planning must be precise and work in real time. Such situations often lead to failures in global path planning. When an unexpected obstacle appears in the robot’s path, it is necessary to re-plan to avoid colliding with the obstacle [21]. Planning in an unpredictable setting is a complicated issue due to the generated map must always be adjusted at every iteration of the path planning algorithm. To offer a complete solution to such a problem, many autonomous systems combine the features of global and local navigation systems [22].

The uncertainty and complications of the path-planning problem in a local environment with obstacles have drawn the interest of many researchers [23] and have been a subject of great interest in recent years. As a result, such path planning algorithms have been thoroughly explored, with several approaches and the solutions presented to solve this problem being as follows:

The artificial potential field (APF) method, invented by Khatib [24], is a traditional path-planning method based on potential energy (gravity, magnetic field, or gravitational). A robot in the coordinate space could travel using this method while being driven by attractive and repulsive fields produced by obstacles and targets. This method is used in the robotics field due to its analytical clarity, real-time performance, and ease of use in determining the shortest path from start to goal positions.

Barraquand et al. have introduced a search-based path-planning method based on the potential field with a direct path search [25]. Despite the many benefits that APF provides due to its simple structure, it has certain drawbacks, such as that the robot can become trapped in local minima based on the obstacle location and the potential field they produce. To address this issue, several studies have proposed various ways to avoid local minima in APF [26].

Virtual field histogram (VFH) [27,28] and enhanced VFH+ [29] are prominent approaches that describe the sensor uncertainty due to spatial data from sensors, similar to APF. VFH and APF will perform efficiently with dynamic and static obstacles in constrained settings. Numerous path planning methods that consider obstacles in unpredictable settings have been discussed in [22,30,31]. Ayawli et al. [32] provided a path-planning method in an uncertain environment based on the Voronoi diagram and computation geometry technique (VD-CGT), employing VD and mathematical modeling, with a narrow rectangular area around the robot used to assess collisions. Ravankar et al. have described collision detection based on virtual obstacles using VD-CGT [33]; however, in [34,35], they show how to use dispersed obstacle knowledge transfer for collision avoidance and path planning in complex environments with multiple robots.

Sampling techniques are remarkable because they use deterministic function sampling to plan using the C-connection space. These functions create a map of the robot’s possible C-space movements [36]. Due to its effectiveness, the sampling-based planning (SBP) methods have received close attention in recent years. The common sampling-based planners are probabilistic road maps (PRM) [37] and rapidly random exploring trees (RRT) [38]. The PRM’s fundamental idea revolves around the distribution of nodes over C-space before connecting them with horizontal lines that form a roadmap graph. By confining the search to a network and interconnecting the free working space, the PRM can effectively determine the shortest paths.

The RRT and PRM algorithms address local minima and high computation periods for pathfinding due to their outstanding practical performance and solid theoretical characteristics [39,40]. A potential function based-RRT* (P-RRT*) integrates APF in RRT* to enhance the convergence of the path into optimal solution [39]. Such algorithms compute many dispersed sample points throughout the free space and link them to establish a tree from which a path is found using a search method using Improved A* [41]. The rapidly exploring random trees (RRT) technique has been frequently used in the literature, but the selection of a specific tree to be expanded is the most critical issue that impacts the overall efficiency of path planning in RRT. Wang et al. [42] have presented revolutionary multi-RRTs based on learning for mobile robot path planning (LM-RRT) in narrow passages.

Bidirectional-RRT [43] uses a bidirectional tree search for faster path planning. Similarly, work in [44] proposed a lazy PRM method, an enhanced PRM that minimizes the frequency of collision tests that occur during pathfinding and, therefore, minimizes the planner’s run time. On the other hand, the work presented in [45] demonstrated how to improve the roadmap’s connectivity by linking previously developed connected components. Probabilistic road maps-star (PRM*) is an improved variant of the initial PRM method, which was proposed by [46]. With such a method, the number of sample nodes determines the connection distance. As the number of samples grows, the connection radius gets smaller, making it easier to move from one place to another. For moving from one node on the generated roadmap to the next, SBP approaches employ a local planner. The planning issue is resolved by finding the shortest route between the start of the roadmap on one side and the roadmap to the goal on the other side. Search-based techniques, such as the Dijkstra and A* algorithms, have been used to discover the shortest paths on a built network. The A* method starts by examining the undiscovered node and has the lowest projected cost [47]. These search plans are fast and can work with maps of various sizes. By analyzing the mechanics of the robotic system, when the design has been generated on the graph, the path smoothing methods can generate a smoother path from start to goal positions [48] such as Infused Tangential Curves (ICT) method [49].

Several authors have studied the advantages of the genetic algorithm (GA) for addressing the planning problems for autonomous robots in complex environments. Some of these algorithms depend on a novel fitness function for controlling the distance, safety, and energy of the robot [50], a knowledge-based GA [51], and adaptive GA [52]. Another way to find a collision-free path is to calculate artificial potential values using GA. This method was initially used in [24] for solving the obstacle-free mobile robot path-planning problem. The robot is considered as an object that is navigating in various surroundings, and the technique is cantered upon the attraction and repulsion forces produced by the goal and obstacles [52].

The authors in work [53] presented a hybrid metaheuristic based on the genetic algorithm–particle swarm optimization (GA–PSO) method for mobile robot path planning in grid maps to determine a feasible path from predefined starting and ending points. In contrast to traditional GA and PSO algorithms, the suggested technique avoids computational complexity and premature convergence. First, the integer factorization problem (IFP) is generated using a hybrid GA–PSO, and then a cubic B-spline method is adopted to provide a near-optimal obstacle-free path. Bi et al. have presented a robot-path-planning approach using fuzzy logic and evolutionary algorithms to lower the complexity of robot path planning during obstacle avoidance in a dynamic environment [54].

The use of deep reinforcement learning (DRL) for robot navigation in environments with unknown rough topography, such as in urban search and rescue (USAR), is investigated by Zhang et al. [55]. In this work, they created an Actor-critic-model-3 (A3C)-based network that employs depth images, elevation maps, and 3D orientation as inputs to identify the best robot navigation movements. The network was trained in a series of simulated 3D environments that have been varied in traversal motion. The experiment’s results reveal that when the rough terrain is unknown, the DRL approach may successfully navigate a robot in an environment to a specified target location.

A detailed study on the computational intelligence (CI) algorithms with the time domains and the environment models that are used for 2D/3D-unmanned aerial vehicle (UAV) path planning was presented by Zhang et al. [56]. The authors analyzed the modeling, optimization criteria, and path planning algorithms for UAV robots and concluded that the common methods to address the path planning of mobile robots are genetic algorithm (GA), particle swarm optimization algorithm (PSO), artificial potential field (APF), and ant colony optimization algorithm (ACO).

Patle et al. [57] have thoroughly examined several mobile robot navigation techniques that are now commonly used in robotics applications. The investigations of classical and reactive approaches were presented in detail. The review compares tabular data and charts for the individual navigational strategies that can be used with specific robotics applications. Seckin [58] has presented a method for robot navigation using the real-life bookmarks arranged in its memory. With this method, the robot utilizes the memorized traveled path with several key points to plan its path from starting to target positions. It uses the laser detection and ranging sensor (LIDAR) for mapping the environment into 2D maps. The robot is then driven on its path using the previously prepared map in its memory. Another method for memorizing the robot’s path through generating a network of reuse paths in planetary exploration has been proposed by Stenning et al. [59]. It expands the visual teach and repeat method that allows the robot to visit and revisit any network nodes. To select the right path, the rapidly exploring random tree (RRT) method is used to effectively plan the robot’s path in such networks. Khan et al. have proposed RNN-based metaheuristic algorithms for obstacle avoidance, robot path planning, and control [60,61]. Table 1 shows a comparison between reviewed methods in mobile robot path planning in terms of algorithms, testing types (simulation or physical), sensors used, obstacle avoidance, and indoor/outdoor applications, as follows:

The contribution of this paper is to present an approach for mobile robot path planning in complicated environments with the presence of obstacles. In circumstances where standard methods fail to offer a solution, such as for small passages, obstacle avoidance, and local minima problems, the generalized laser simulator (GLS) can overcome such associated path-planning problems. Furthermore, the algorithm presented an optimized version that will generate pathways with lesser nodes and a greater success rate while being computationally efficient. It is an enhancement of the laser simulator method, which has been successfully adopted in many works [62,63,64,65] and an extension of [1] for avoiding obstacles during path planning.

## 3. Generalized Laser Simulator (GLS) Algorithm

The mobile robot path-planning process involves moving a mobile robot from a start position and navigating it through possible successive points until it reaches the target position in a known or unknown environment. The robot must avoid colliding with objects and must optimize the path from the start to the goal.

The path planner’s environment (or configuration space) is divided into two sections, namely, free space and space with obstacles. The robot’s predefined start and target points are positioned in the free space. The robot’s path-planning objective is to find a fixed set of possible points to navigate the robot from the start to the target. When there are several paths between the start and goal positions, mobile robot path-planning algorithms are employed to find the optimum path according to some specific decision variables, such as the path distance, path smoothness, or obstacle avoidance. This paper presents a new path-planning algorithm for determining the best path between a predefined start and a target location in the environment.

A path’s quality is determined by several factors, namely (i) path distance, (ii) path smoothness, and (iii) path reliability. Therefore, this paper presents the path-planning problem in 2D maps with circular-shaped obstacles and no connection to the surrounding space. The robots are also considered points, with their sizes used to calculate the confidence radius around the obstacles.

The proposed mobile robot path-planning method GLS will determine an optimal path for the mobile robot from a start to a goal position with some random obstacles dispersed in a working space. As a result, the robot will safely move from the start to the target nodes while avoiding static or dynamic obstacles. The primary advantage of this algorithm is its capability to locate the best and smooth path to the destination while avoiding obstacles.

In this work, the path-planning problem is broken down into three parts: avoiding obstacles, searching for a goal, and finding an optimal path for the mobile robot in both known and unknown environments. The ability to apply this algorithm to local and global navigation in the presence of obstacles in known and unknown environments is one of its primary features.

### 3.1. Modelling of Workspace

The workspace configuration for solving robot path-planning problems using the presented algorithm is described in detail. Workspace-configuration-based map generation is an essential part of the path planning process. It is required to acquire map information about the workspace environment before planning a robot path and determine a feasible path for the mobile robot to move from the start until the end of its movement.

A two-dimensional f (x, y) function is used on each map. Each pixel has a single value, representing the intensity of the light at that particular position in the image. The function’s value represents the grayscale intensity at each (x, y) location. The lowest grey level is 0, and the highest grey level is 255.

#### 3.1.1. Modelling of Workspace for Global Path Planning

To test the GLS path planning algorithm, a 2D map is used to determine the feasible path for the mobile robot. In this map, polygons will represent the robot’s surrounding environments on a 2D axis. A series of points in GLS will be generated as waves in all directions between the start and goal positions to achieve a feasible path within a short search time.

After developing the mobile robot workspace environment, four static circular-shaped obstacles are placed in the workspace at different locations. Equation (1) gives the locations of the obstacle’s center.

(1) $$x_{obs}=\lfloor x_{i}\rfloor \;\mathrm{and}\;y_{obs}=\lfloor y_{i}\rfloor$$

where *x_i_* and *y_i_* are the obstacle’s center positions. To find suitable space for the obstacles, the free and occupied spaces (C) were calculated. Equation (2) is used to determine the obstacle space border.

(2) $$\;\;\;\;\;\;\;\;\;\;\;\;\;\;\;\;\;\;\;\;\;\;\;\;\;\;\;{\left(x-x_{obs}\right)}^{2}+{\left(y-y_{obs}\right)}^{2}=r^{2}$$

where *r* is the obstacle’s radius. Equation (3) can be used to find the total space occupied by the obstacles.

(3) $$\;\;\;\;\;\;\;\;\;\;\;\;\;\;\;\;\;\;f\left(g\right)=\sum_{i=0}^{N}\pi r_{i}{}^{2}$$

where *N* is the obstacles’ number.

A Euclidean representation is used to depict the workspace. The obstacles’ borders are considered the same as the environment’s borders.

In the case of dynamic obstacle avoidance, the generated obstacle is set to travel randomly (top/bottom or left/right). The distance between the obstacle and the nearest borders or boundaries determines the direction of obstacle movement. Equation (4) determines the obstacle’s index distance from up, down, left, and right boundary positions. Finally, the mobile robot chooses the direction of movement using Equation (5). It always decides to travel to the border farthest from where it started.

(4) $$\mathrm{obtacle}\;\mathrm{index}\;\mathrm{distance}\;{\mathrm{id}}_{o}=\{\begin{array}{l} (x_{\mathrm{obs}},y_{\mathrm{obs}}+1:\mathrm{end})\;\;\;\;\;\;\;\;\;\;\;\;\;\mathrm{right}\;\mathrm{border}\\ (1:x_{\mathrm{obs}},y_{\mathrm{obs}})\;\;\;\;\;\;\;\mathrm{upward}\;\mathrm{border}\\ (x_{\mathrm{obs}},1:y_{\mathrm{obs}})\;\;\;\;\;\;\;\;\;\;\;\;\;\mathrm{left}\;\mathrm{border}\\ (x_{\mathrm{obs}}+1:\mathrm{end},y_{\mathrm{obs}})\;\;\;\;\;\;\;\;\;\;\;\;\;\;\;\;\;\;\mathrm{downward}\;\mathrm{border} \end{array}$$

(5) $$Direction\;of\;movement=\{\begin{array}{l} 1\;\;\;\;\;\;\;\;\;\;\;\;\;upward\\ 2\;\;\;\;\;\;\;\;downward\\ 3\;\;\;\;\;\;\;\;\;\;\;\;\;\;\;\;\;\;\;\;\;\;left\\ 4\;\;\;\;\;\;\;\;\;\;\;\;\;\;\;\;\;\;right \end{array}$$

Equation (6) is used to determine the movement of the obstacles.

(6) $$\mathrm{Obstacle}\;\mathrm{movement}=\{\begin{array}{l} (x_{\mathrm{obs}}-\mathrm{radius},y_{\mathrm{obs}})\;\;\;\;\;\;\;\;\;\;\;\;\;\mathrm{if}\;\mathrm{direction}\;\mathrm{is}1\\ (x_{\mathrm{obs}}+\mathrm{radius},y_{\mathrm{obs}})\;\;\;\;\;\;\;\;\;\;\;\;\;\mathrm{if}\;\mathrm{direction}\;\mathrm{is}2\\ (x_{\mathrm{obs}},y_{\mathrm{obs}}-\mathrm{radius})\;\;\;\;\;\;\;\;\;\;\;\;\;\mathrm{if}\;\mathrm{direction}\;\mathrm{is}3\\ (x_{\mathrm{obs}},y_{\mathrm{obs}}+\mathrm{radius})\;\;\;\;\;\;\;\;\;\;\;\;\;\mathrm{if}\;\mathrm{direction}\;\mathrm{is}4 \end{array}$$

The dynamic obstacle (black circle) will appear randomly in the working environment. Distance between the obstacle and each of the four (top, bottom, left, right) border is calculated, and the maximum displacement between them is chosen as the set realistic goal.

#### 3.1.2. Modelling of Workspace for Local Path Planning

This section describes how to implement GLS algorithm in both outdoor and indoor settings. The mobile robot is designed to navigate the environment in real time, detecting and identifying boundaries until it arrives at its predetermined target point.

A wheeled mobile robot (WMR) will be used to evaluate the generalized laser simulator to find the path from start to endpoint based on the acquired data from a camera. The camera will be used with a suitable image processing algorithm to create a 2D map of the area.

The algorithm has been implemented in a platform with a ready control system in which the path planning will calculate the heading angle of the robot, which will later be performed using the control system, based on GLS. The processor used in this research is interface-free controller (IFC), which uses parallel data manipulation, which helps to accelerate the robot’s performance. The GLS algorithm is used to find the robot path while developing a local map based on live-streaming video. The approach is practically identical to that developed in Section 3.1.1, except that the robot communicates with a live video rather than a single image.

The video is acquired through a camera with high resolution and analyzed in MATLAB software with image processing tools that can capture and process some frames of the video in real time.

**Figure 1:**
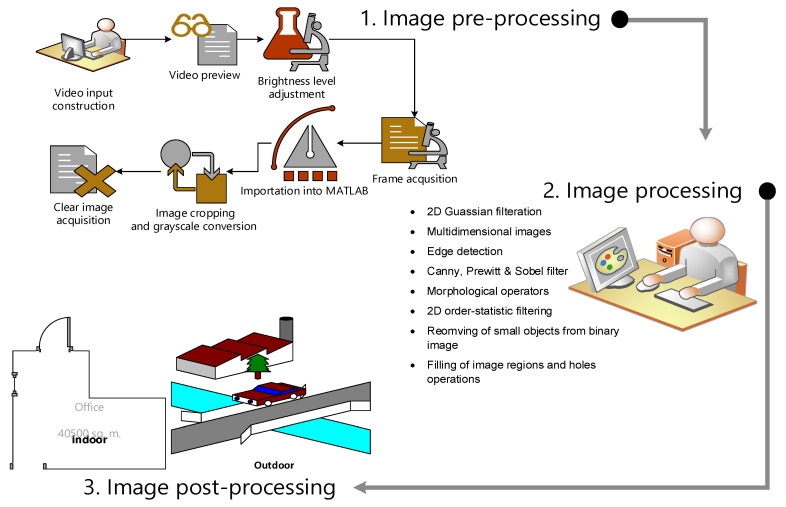
Image processing steps.

The image-processing algorithm has been developed for extracting image frames from the streaming video, applying several operations to create the local map image and performing other computations for road border detection and image processing. There are three main steps in the image processing algorithm:

- Image preprocessing for preparing the images is shown in Figure 1.
- Image processing and generating a local map for the robot’s working environment. This constitutes processes that allow for the extraction of road borders from images and the removal and filtering of noise.
- Post-processing algorithms for local path planning.

The image post-processing includes the use of GLS to find the optimum path of the robot, as illustrated in Figure 1.

The proposed algorithm has been encoded in MATLAB and converted to Visual C Sharp (VC#) through the com server automation server, which has the driver for dealing with IFC cards. The data and signals are exchanged between the cards and Visual C#. Then, the pulse width modulation (PWM) signals enable the movement of the motors coupled with wheels through the motor driver. The GLS path planning will find the next position of the robot through generating waves that will intersect with the borders and obstacle border; then, it chooses to move through the middle. To move from the current position to the planned next position, the controller will calculate the heading angle and move the robot towards the next planned position.

### 3.2. Formulation of GLS

The path planner’s primary aim is to determine the most efficient way for a mobile robot to navigate while avoiding obstacles during autonomous navigation from a start position to a target position in an environment.

This section explains how to determine a feasible collision-free path using the proposed generalized laser simulator algorithm. This algorithm is an enhancement of the laser simulator method, which has been successfully adopted in many works [60,61,62,63] and an extension of [1] for avoiding obstacles during path planning.

**Figure 2:**
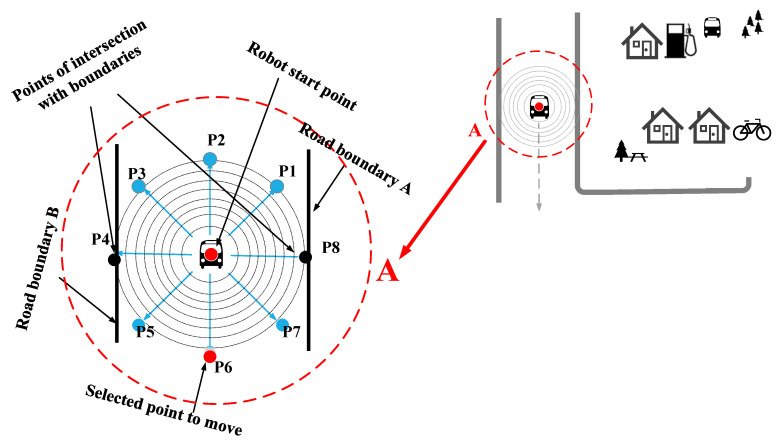
GLS method process.

In [1], the GLS algorithm has been utilized to determine the path within the restricted environment. It finds the borders of environments and determines the next position of robot through generation of a series of points like waves from the beginning until the goals positions. The waves will be continuously generated until detection of the boundary of environments as shown in Figure 2.

Using the concept of the shortest path to the goal and the longest distance between the mobile robot and the nearest border, the GLS algorithm can determine a feasible path from the start to the target point. The shortest distance process analyses the candidate points and determines which has the least distance to the target. Equations (7) to (10) are used to calculate the probability of the preferred point. The theory of negative probability was adopted to determine the most feasible site to travel to.

(7) $$Distance,\;D_{i}=\sqrt{{\left(x_{goal}-x_{p_{i}}\right)}^{2}+{\left(y_{goal}-y_{p_{i}}\right)}^{2}}$$

(8) $$d_{min}=\min_{i=1}^{8}D_{i}=\min\left(D_{1},\;D_{2},\ldots \ldots ,D_{8}\right)$$

(9) $$P_{prob}=\frac{D}{\sum_{i=1}^{8}D_{i}}P_{prob}\min=\frac{d_{min}}{\sum_{i=1}^{8}D_{i}}$$

(10) $$P_{prob1}=1-P_{prob}$$

**Figure 3:**
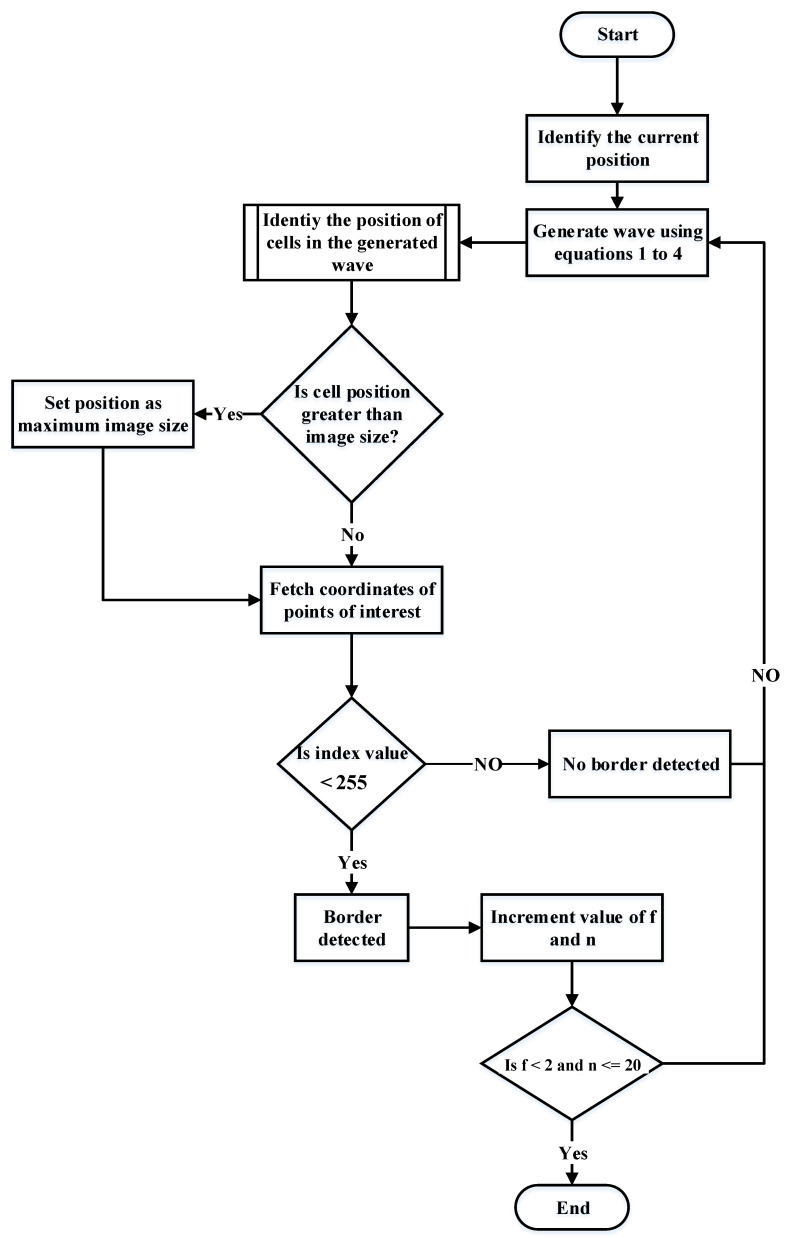
Flow chart of GLS working procedure.

The second parameter focused on the distance of an index from a border. From the initial point, the total distance between the target place and the nearest boundary in the right, left, top, and bottom directions is calculated, as seen in Figure 3b. The theory of negative probability is also used in this process, which can be seen in Equations (8)–(10), in which the lowest probability is chosen.

The distance between the eighth point and the border in all directions is calculated using Equation (11):

(11) $$x_{di}=x_{pi}+rcos\left(\gamma \right),y_{di}=y_{pi}+rsin\left(\gamma \right)\;\mathrm{and}\;D_{bi}=\sqrt{x_{di}{}^{2}+y_{di}{}^{2}}$$

where *r* is the radius of the circle intersected with the border. γ is equal to 45°.

The probability of the maximum distance is calculated using Equations (12) and (13):

(12) $$d_{b\;max}=\max_{i=1}^{8}D_{bi}=\max\left(D_{b1},\;D_{b2},\ldots ,\;D_{b8}\right)\;$$

(13) $$P_{prob2}=\frac{D_{b}}{\sum_{i=1}^{8}D_{bi}}\;\mathrm{and}\;P_{prob2\;}max=\frac{d_{b\;max}}{\sum_{i=1}^{8}D_{bi}}$$

Equations (12) and (13) calculate the best candidate path point for the mobile robot based on the probability summation. The best-suited candidate point for the path is calculated using Equations (14) and (15):

(14) $$T=\sum_{i=1}^{2}P_{prob\left(i\right)}$$

(15) $$Q=max_{i=1}^{8}T_{i}=\max\left(T1,\;T2,\;\ldots ,\;T8\right)$$

An additional optimization technique is used to find the shortest path. The optimized GLS would lower the number of the GLS path points between start and goal positions, resulting in an optimum path.

Figure 3 shows the steps of generating GLS algorithm from start to goal position.

### 3.3. Obstacle Avoidance

A mobile robot will approach the target point from any starting position in a collision-free environment. In case of the occurrence of an obstacle, the mobile robot should detect it from a significant distance and seek to avoid it. As a result, the mobile robot can avoid the obstacle by adjusting its present trajectory to a different one within the free workspace available in the environment. Obstacle avoidance can be handled for a static or dynamic obstacle.

**Figure 4:**
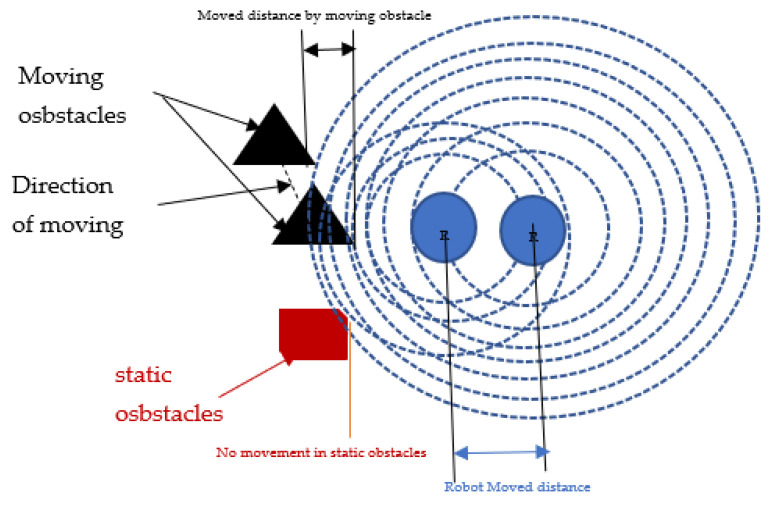
Static and moving obstacle detection using GLS.

The GLS algorithm considers the boundaries of the static obstacles, such as the borders of the environment. However, for dynamic obstacles, the direction of moving obstacles will be determined through sending waves that are continuously intersecting with moving obstacle borders. By comparing the position of the robot and the obstacle borders in the next generation of the waves, we can find whether this border is related to static or moving obstacles as it moves near or far from the next generated waves of moving obstacles, as shown in Figure 4.

*Total moved distance* = *robot/wave original movement* ± *obstacle movement*

If the total moved distance is equal to robot/wave original movement, then it is a static obstacle; however when the total moved distance is smaller or larger than the robot/wave original movement, it is a moving obstacle.

#### 3.3.1. Static Obstacles Avoidance

**Figure 5:**
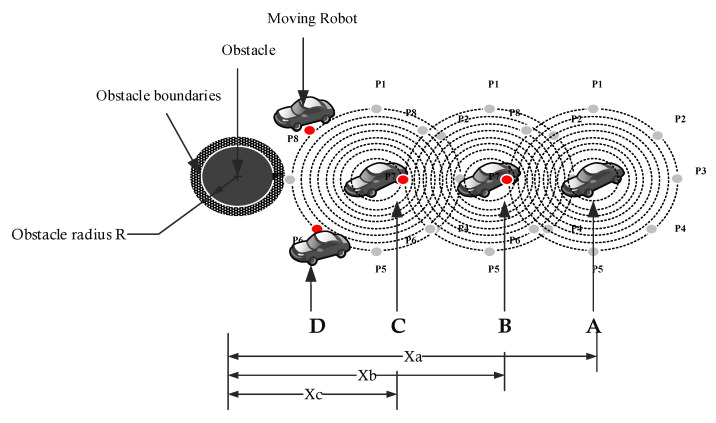
Static obstacle collision avoidance.

Let us consider the situation given in Figure 5 to illustrate static obstacle avoidance. As shown in Figure 3, at point A, the mobile robot generates waves to detect borders at iteration t, where it chooses the point P7 as the next preferred point to move to. As the mobile robot moves to an updated position (see point B) and approaches the obstacle borders at iteration t + I, a similar situation is experienced. At point C, the borders of the obstacle intersect with the vertices of P7. Hence, the robot explores the workspace (either P8 or P6) for the following position while avoiding collisions, and then, the mobile robot would either move up or down (see point D). The obstacle’s boundary is regarded as a border in static obstacle avoidance.

#### 3.3.2. Dynamic Obstacle Avoidance

When the robot approaches a moving obstacle, it instantly determines its movement direction. Then it automatically switches to the next position and escapes the obstacle depending on the robot’s current motion.

The kinematics of the robots and obstacle(s) were not considered in the proposed method. The detection of obstacles happens during the execution of GLS in dynamic obstacles; however, the feasible path is generated in the GLS optimization process.

The goal of the optimization process is to determine the most efficient path that the robot will navigate without obstacle collision. The optimization process begins with identifying the present and selected points’ positions. Then, using an ascending approach, all x values of the selected positions are organized into a vector X. Similarly, y values are structured as a vector Y, having ascending values in the y direction.

The minimal distance between other points and the goal point is calculated by considering the distance between the goal and starting points. If the goal is higher than the starting point, it is taken in incremental order. Alternatively, if the goal is lower than the starting point, it is taken in the opposite order. As a result, the coordinate values in the x–y plane of the selected points from the start position have been ordered in this manner—in either increasing or decreasing order needed to reach the goal position. Based on varying circumstances, the optimal location range for the robot to move was determined to be fifteen pixels.

The current and newly selected positions were then evaluated to see whether they matched or not to such a point. If they did not match, the possibility of moving to the next preferred point without walking across a border or obstacle was investigated.

**Figure 6:**
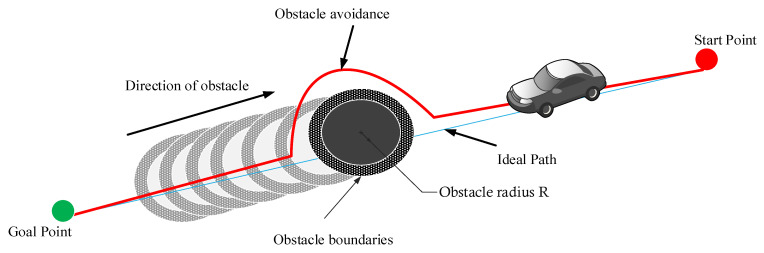
Dynamic obstacle collision avoidance.

Because the obstacles are believed to be circular, the robot deviates from its path by switching its motion to rotational movement using Equation (16), as seen in Figure 6.

(16) $$x_{next}=x_{current}+radius\times \cos\emptyset$$

(17) $$y_{next}=y_{current}+radius\times \sin\emptyset$$

where ∅ represents the half-circle arc angle $\left(0<\emptyset <\frac{\pi }{2}\right)$.

### 3.4. Experimental Settings

In this work, the proposed method was tested in several settings and scenarios to demonstrate its feasibility for finding the correct path of the robot within the surrounding environment. The source maps have a 500-by-500-pixel resolution, and all codes were written in MATLAB (MathWorks Company, Kuala Lumpur, Malaysia) R2014b on a 64-bit Win 64 pc with an Intel (R) Core (TM) i5 2450 M processor.

#### 3.4.1. Investigation of GLS in Global Path Planning

The proposed method’s quality was assessed in some workspace settings with obstacles configuration. Ten different randomized environments were developed and simulated. Each environment was tested 50 times in the proposed algorithm. Each run’s path search time and distance were recorded, and the average mean with standard deviation values was calculated. The start and target locations were placed at different locations in each map’s free space. The algorithm produced safe, short-distance paths that took a reasonable amount of time.

#### 3.4.2. Investigation of GLS in Local Path Planning

**Figure 7:**
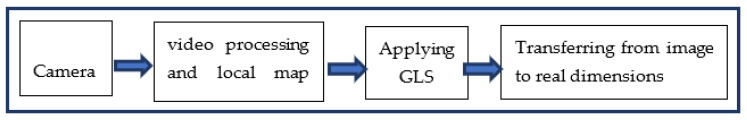
Processes of determining the position of the robot.

To draw a local map with x and y dimensions in local path planning, one must transfer all image pixels into an actual dimension using the camera transformation, as explained in detail in [59]. However, this process will take a long time and slow the processing time of the whole system. Thus, only the robot’s position will be transferred from the image plane to the real world dimension. Figure 7 shows the whole process of determining the robot’s position.

### 3.5. Performance Metrics

To evaluate and measure the performance of the proposed algorithm, the following parameters are used:

#### 3.5.1. Total Search Time (ST)

Each algorithm’s total search time is measured in seconds. This parameter is important since the best algorithm must generate paths in a fast and effective manner. The search time is measured using a timer coded in MATLAB. A timer event is triggered whenever the counter clock strikes the timer period. The initialization resets the counter and sets the timer period. If the period is set to zero, the timer will not run; instead, the timer will be increased with every clock increment.

The standard deviation (SD) is a statistical metric for assessing precision and repeatability. Its value represents how far the individual variables’ values deviate from the mean value. The search times of each algorithm’s relative SD are calculated using Equation (17):

(18) $$SD=\sqrt{\frac{\sum_{i=1}^{n}{\left(ST_{1}-ST_{n}\right)}^{2}}{ST_{n}}}$$

where $ST_{i}$ Is an algorithm’s *i*th search time during the simulation in a specific experiment.

#### 3.5.2. Path Cost (PC)

The path cost (PC) is the distance that the robot travels from a start point to a specified endpoint. It is the distance of the total paths covered during the search measured in the unit of cells. This information is crucial since the mean path cost is directly proportional to the path’s length.

Similar to the search time, the standard deviation of each path’s cost is calculated using Equation (18).

(19) $$\;\;\;\;\;\;\;\;\;\;\;\;\;\;\;\;\;\;\;\;\;\;\;SD=\sqrt{\frac{\sum_{i=1}^{n}{\left(PC_{i}-PC_{n}\right)}^{2}}{PC_{n}}}\;\;\;\;\;\;\;D=\sum_{i=1}^{Path\;size-1}Distance\left(\;Path_{i},Path_{i+1}\right)$$

where $PC_{i}$ is an algorithm’s *i*th path cost in the unit cell, and ***D*** is the total path.

#### 3.5.3. Path Smoothness

Path smoothness can be accomplished by assessing the path pattern outlooks generated by path planning methods, indicating whether the robot trajectory has a zigzag or not.

## 4. GLS Implementation in Local and Global Path Planning

Several experiments have been conducted to test the proposed algorithm in local and global environments as follows.

### 4.1. Investigation of GLS in Global Path Planning

The proposed approach is tested with obstacles to identify a feasible path from the defined start location to target locations. In addition, the performance of the proposed method with and without the presence of obstacles is compared.

The results of the path determination in both static and dynamic obstacles are as follows:

#### 4.1.1. Static Obstacle

**Figure 8:**
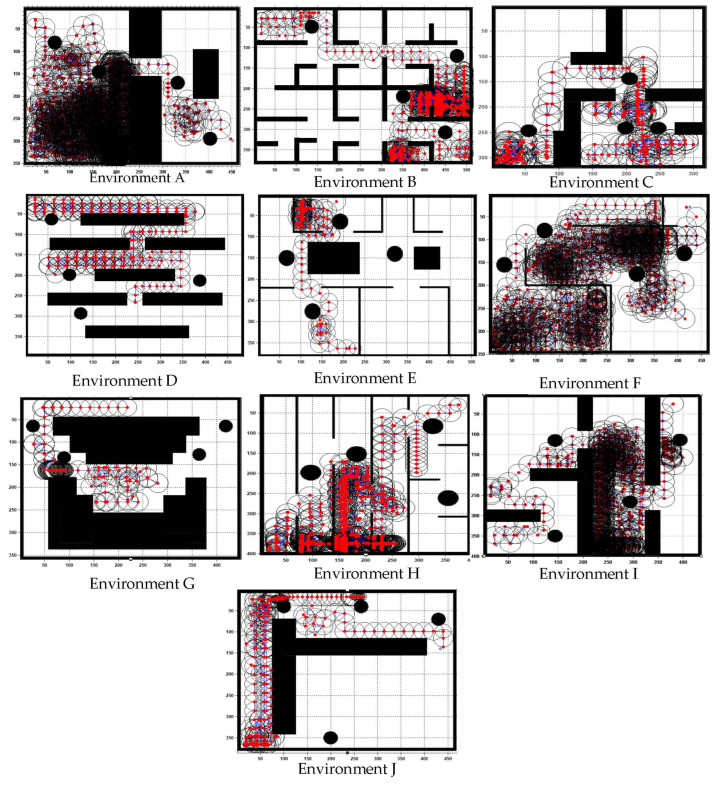
Results of obstacle avoidance GLS—first stage (wave generation).

The working environment is divided into small pixels. Each black pixel can depict either the border of the environment or an obstacle-filled space. Four spherical obstacles were placed across the workspace. The simulated results of 10 workspace settings with four static obstacles placed randomly at positions x(g i), y(g i) are shown in Figure 8. The starting and target point coordinates are (xs, ys) and (xg, yg). The proposed approach has been tested to select the best path from the start to a target point.

**Figure 9:**
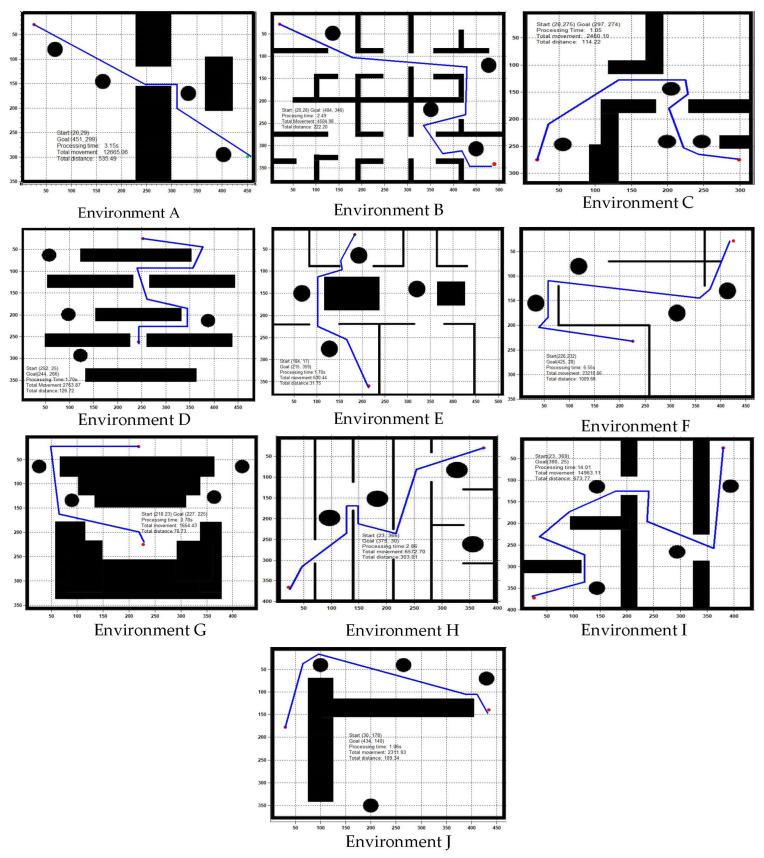
Final path for obstacle avoidance after GLS—second stage (optimization).

**Figure 10:**
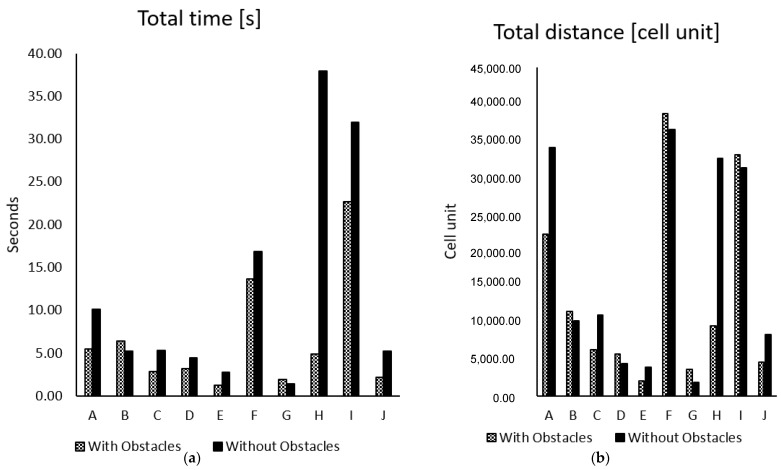
Total search comparison for static obstacle avoidance: (**a**) time, (**b**) distance.

The previously generated paths in Figure 8 are not optimal in terms of time cost, path smoothness and path cost. To decease the overall path cost, the algorithm in stage 1 has been further enhanced and the resulted path of robot is shown in Figure 9. The path and costs for all runs of experiments have been presented in Figure 10. Such optimization has led to short and safe paths with low time cost. In the occurrence of obstacles, the search time for pathfinding is lower than when there are no obstacles. This is due to the algorithm having to detect environment/obstacle borders or reach its maximum wave generation of 20, as discussed above. The calculated results for search time measured for 50 trials in each of the ten different environments are shown Figure 10.

It can be noted that the total time and distance in environments with obstacles are lower than in environments without obstacles, as shown in Figure 10. This is due to the fact that the obstacle’s boundary is regarded as a border similar to other environment borders. In the absence of obstacles, the GLS algorithm must explore more paths during the border detection phase until it detects borders or reaches its maximum wave-generating capacity, which results in additional steps and distance being generated, costing increasing amounts of time. On the other hand, the presence of obstacles shortens the time and distance required to find borders.

Figure 10’s graphs demonstrate that the presented method can effectively guide the Robot toward the target in a complicated environment while avoiding collision with static obstacles.

**Figure 11:**
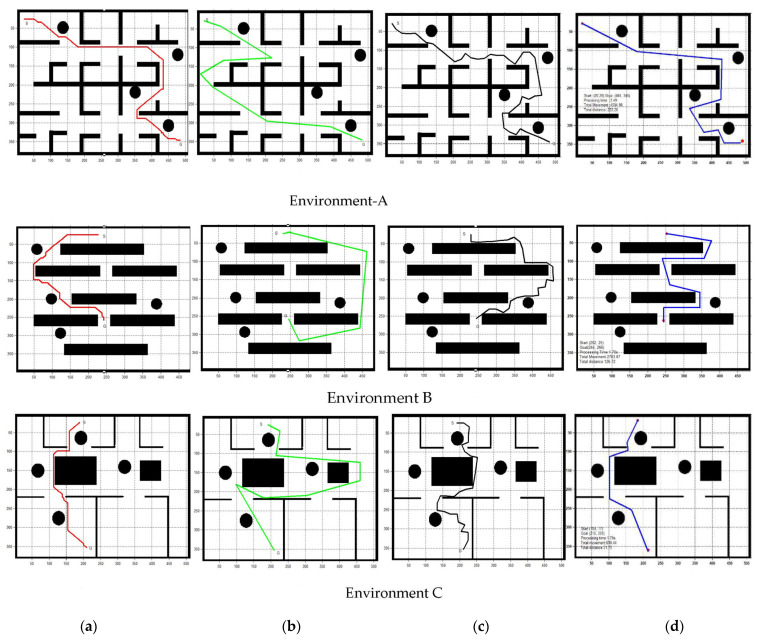
Comparison between (**a**) A*, (**b**) PRM, (**c**) RRT, and (**d**) GLS.

The performance analysis comparison between the GLS algorithms and three other algorithms (A*, RRT, and PRM) for three different environments with four randomly distributed static obstacles was presented in Figure 11. It was decided to compare A*, RRT, PRM, LS, and GLS as their natures depend on exploring the environments through generating trees (RRT), lines (PRM, LS, GLS), and distance calculation (A*) to find the correct position of the robot. Therefore, they have similar procedures of finding paths from the start to the goal position. Thus, one can effectively compare them.

In addition, A*, RRT, and PRM are among the most popular and most frequently used algorithms that have proven to be reliable for solving path-planning problems. For LS, the GLS algorithm is an extended version of the LS algorithm, so the performance comparison is necessary to see its effectiveness in comparison with the original LS algorithm.

**Figure 12:**
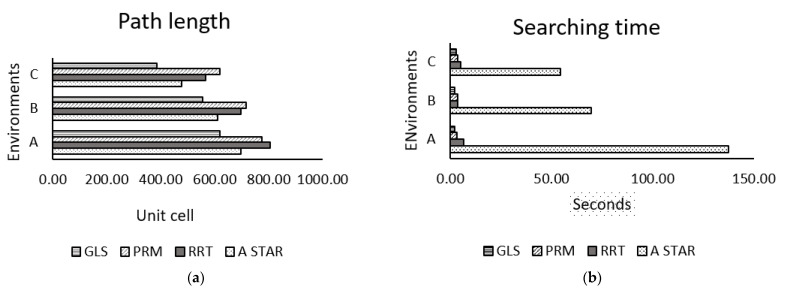
Mean values of the fifteen trials in Table 2 and Table 3 for GLS, PRM, RRT, and A* algorithms in environments A, B, and C: (**a**) path length, (**b**) searching time.

Table 2 and Table 3 give the tabulated values of total distance and searching time of PRM, RRT, A*, and GLS algorithms, which are recorded for 15 trials of three environments—A, B, and C—as in Figure 11. The mean value for the path cost and searching time of the fifteen trials are graphically compared in Figure 12.

Figure 12a shows the graphical comparison of the path length between PRM, RRT, GLS, and A* methods for three environments (A, B, and C) with the same start and goal positions. It is clearly seen that the path length of the GLS algorithm is shorter compared to the PRM, RRT, and A* algorithms. Figure 12b displays the graphical comparisons of processing time in seconds of PRM, RRT, GLS, and A* methods for the same three environments’ start and goal positions; A*’s running time is much higher compared to the other three methods, while the proposed GLS algorithm shows the shortest running time.

**Figure 13:**
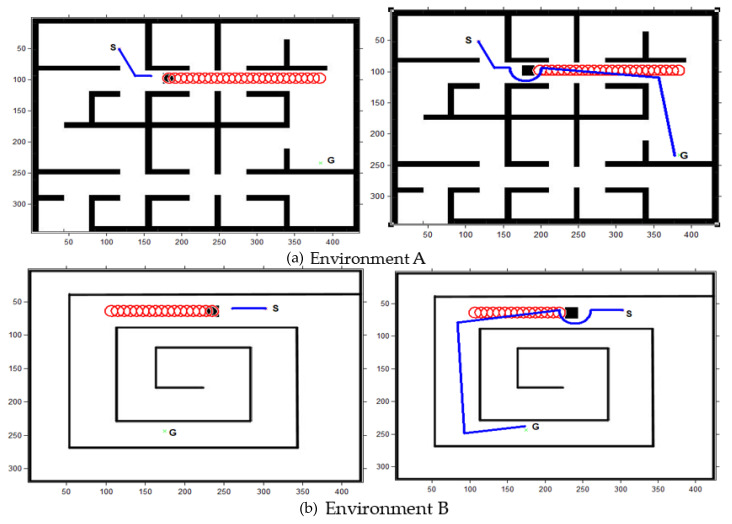
Dynamic obstacle avoidance: (**a**) Environment A, (**b**) Environment B.

For dynamic obstacles, the performance of GLS is better than the original LS. Regarding time average as in Figure 13a, GLS is performing faster than LS by 5.5 times. Regarding distance average, as in Figure 13b, GLS presents a shorter path than LS by 1.5 times. Therefore, it can be concluded that the GLS has outstanding performance in both path and time costs for pathfinding with obstacle avoidance compared to the other algorithms in dynamic obstacles. In addition, the path of GLS is much smoother than the RRT algorithm, as shown in Figure 11. As shown in Figure 11, RRT and PRM have intersected with all environment borders, which means that their capability to move through small passages is too low. However, A* and GLS have intersected with only one environment, as in Figure 11a,d (Environment C). Local minima are measured by path length, which is shorter in A* and GLS and high in RRT and PRM.

#### 4.1.2. Dynamic Obstacles

The subsequent motion must be adjusted accordingly when the robot approaches a dynamic obstacle to avoid the obstacles intelligently. The experimental result of dynamic obstacle avoidance in two different environments is shown in Figure 13.

### 4.2. Investigation of GLS in Local Path planning

The generalized laser simulator algorithm has been tested in both indoor and outdoor settings as follows:

#### 4.2.1. Indoor Results

The images sequences of the camera’s video is utilized to accomplish the indoor navigation of mobile robots. The sampling time for processing is the slowest sensor (camera and odometry). The slowest device is odometry, which has 800 pulse/rotation and 100 rpm from a DC motor. The time for giving one pulse is 2.083 ms, and that for complete rotation is 1.66 s. The processing time for the data is within 1 ms. Therefore, the total processing time for receiving one signal is 3.08 ms.

**Figure 14:**
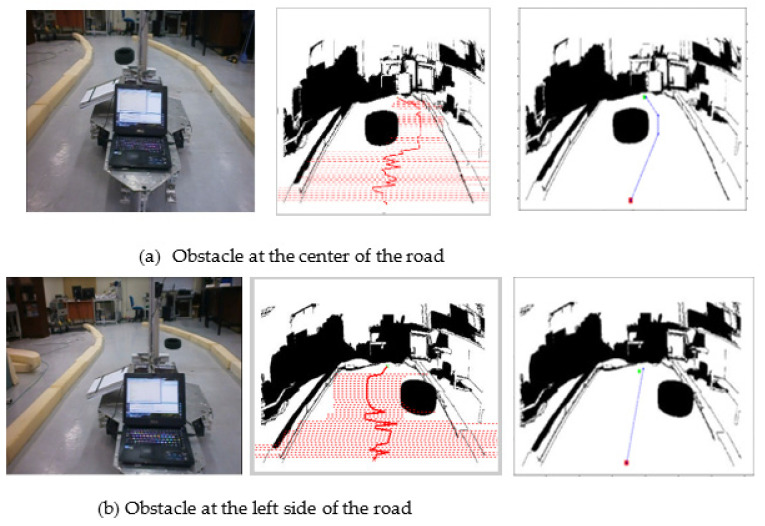

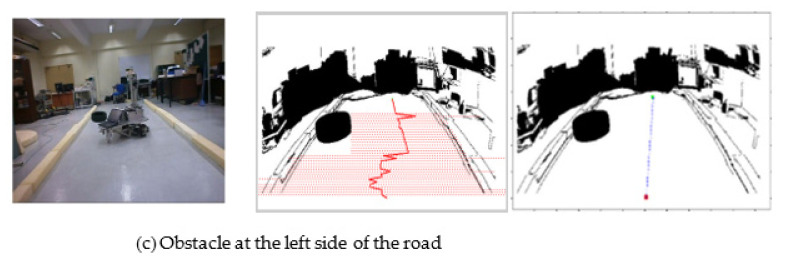
Indoor navigation with obstacle on the left side of the road. (**a**) Original image; (**b**) applying LS algorithm; (**c**) applying GLS algorithm.

The pre-processing and processing techniques are applied to the image frames. The GLS algorithm examines each image frame’s pixel value in the environment borders. The findings of indoor navigation in Figure 14 demonstrate that the image processing algorithm is able to clearly build a map of the environment with obstacles from a series of image frames. Figure 14 represents the post-processing path results of the proposed GLS with obstacle positions to the center, left, and right of the robot’s path. Figure 14a shows the original image of the wheel mobile robot, while Figure 14b,c show the final paths generated using the LS and GLS algorithms, respectively.

**Figure 15:**
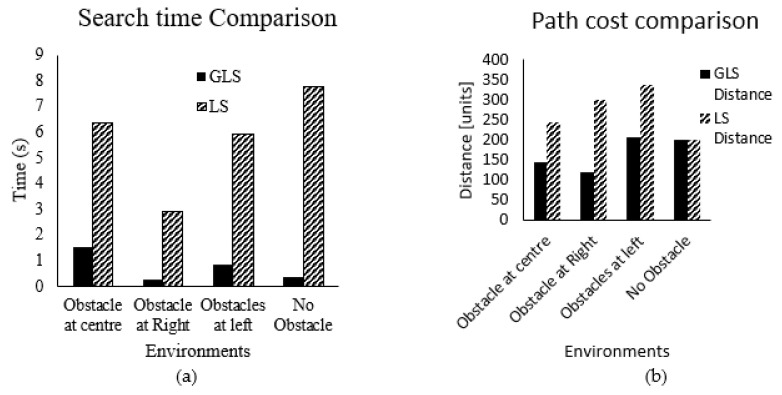
Comparison between GLS and LS in indoor environment: (**a**) search time, (**b**) distance.

Figure 14 provides a graphical representation of the path findings, but it is difficult to determine which method is the best. Figure 15 shows a graphical comparison of path cost and running time between LS and GLS. In comparison with the original LS method, the proposed algorithm outperforms the LS algorithm in terms of path and time costs and path smoothness.

#### 4.2.2. Outdoor Results

**Figure 16:**
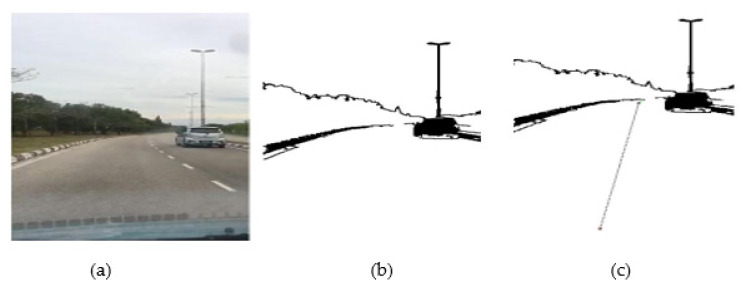
GLS with obstacles in the road. (**a**) Original image; (**b**) image from pre-processing; (**c**) applying GLS algorithm (dash line).

A mobile robot was operated in a complex outside roads with partial maps where it was required to deal with unexpected obstacles spotted along the way. In these situations, the GLS-based path-planning algorithm must be able to handle path planning in such a partial environment and employ efficient representations. The GLS algorithm has been implemented on a real road at the University Malaysia Pahang Pekan campus, as shown in Figure 16a. The image processing algorithm enables the robot to find the borders of the roads and obstacles, with capability to eliminate the other parts of the road, as shown in Figure 16b. From Figure 16c, it can also be seen that the algorithm can effectively traverse the outdoor environment.

The GLS algorithm can find a path even in a situation in which a border is not detected. As shown in Figure 16, some borders of the road in the bottom of figure are missed. However, GLS is able to find the path. This is due to the fact that in a situation in which the GLS algorithm cannot detect any border, it will stop at the 20th generated circle and determined the next point to move. The absence of borders will only increase the path and time costs, which can be observed in Figure 16 and Table 4.

A comparison between GLS and other algorithms (A*, PRM, RRT, and LS) in both global and local path planning in terms of path cost, search time, and path smoothness is illustrated in Table 4. It can be clearly seen that GLS has the best performance in comparison with all other algorithms, as has been previously explained.

## 5. Conclusions

Path planning is a critical problem in robotics, especially for mobile robots that are working in challenging environments. Artificial Potential field, Sampling-based planner’s methods, and probabilistic roadmap algorithms have been frequently adopted for many robot applications. However, such methods struggle from a narrow passage problem, resulting in an unconnected graph due to the random selection of nodes. The problem is solved by expanding the number of nodes but at the expense of increasing the processing costs, which impacts real-time performance.

This paper presented the generalized laser simulator algorithm for path planning with obstacle avoidance in global and local environments. The implementation of the proposed algorithm in indoor and outdoor environments has been discussed and presented. A camera with suitable image processing algorithms has been used to extract the environment’s features and develop a local mapping for the environment. The GLS algorithm is utilized to plan the path within the developed local maps. The path is modeled, and a collision-free path is generated within its environment.

The proposed algorithm has been implemented in global and local path planning with static and dynamic obstacles in different scenarios. The results have verified that the proposed method can effectively avoid global and local path planning obstacles while searching for the shortest path.

In contrast with the PRM, RRT, laser simulator, and A* algorithms, GLS presents the best path and time costs with a piecewise linear, smooth path.

As future work, the metaheuristic algorithms can be used to speed up the path-planning process of the GLS algorithm through training the path well. For training the paths, one can manually choose as many random points as desired. Later, we try to find paths for each possible point combination and store their details. When running the proposed algorithm, if any trained data exists, it will use such trained data and process the path accordingly. If no trained data exist, then it will process normally with GLS. However, in a situation in which the selected data point is near the trained data points, the result is expected to be fast and very accurate.

## Figures and Tables

**Table 1:** Illustrates a comparison between reviewed works.

Author Year	Algorithm	Static Obstacle	Dynamic Obstacle	Mapping	Online Path Planning	Indoor/Outdoor	Type of Test
Zhang 2018	DRL	Yes	Yes	3D	No	-	Simulation
Chao 2018	GB-RRT	Yes	No	2D	No	-	Simulation
Shum 2015	OUM-BD	Yes	No	2D	No	-	Simulation
Zhang 2012	Multi-objective PSO	Yes	No	2D	No	-	Simulation
Bakdi2017	GA/AFL	Yes	No	2D	Yes	Indoor	Simulation/Experiment
Han 2017	SPS/PI FLP	Yes	No	2D	No	-	Simulation
Muhammad 2021	GLS	Yes	Yes	2D	Yes	Indoor/Outdoor	Simulation/Experiment
Khatib 1985	APF	No	Yes	2D	Yes	Indoor	Simulation/Experiment
Barraquand 1992	PF	Yes	Yes	2D	No	-	Simulation
Cetin 2012	APF	Yes	No	2D	No	-	Simulations
Borenstein 1991	VFHV	Yes	No	2D	No	Indoor	Simulation/Experiment
Ulrich 2000	VFH*	Yes	No	2D	Yes	Indoor	Simulation/Experiment
Ulrich 1998	VFH+	Yes	No	2D	Yes	Indoor	Simulation/Experiment
Ravankar 2020	VFH+	Yes	Yes	2D	Yes	-	Simulation
Tuncer 2012	GA	Yes	Yes	2D	No	-	Simulation
Ayawli 2019	VD/CGT	Yes	Yes	2D	No	-	Simulation
Ravankar 2019	VD/CGT	Yes	Yes	2D	Yes	Indoor/Outdoor	Simulation/Experiment
Ravankar 2017	VD/CGT	Yes	Yes	2D	Yes	Indoor/Outdoor	Simulation/ Experiment
Ravankar 2017	VD/CGT	Yes	Yes	2D	Yes	Indoor/Outdoor	Simulation/Experiment
Qureshi 2016	P-RRT*	Yes	No	2D	No	-	Simulation
Fu 2018	Improved A*	Yes	No	2D	Yes	Indoor	Simulation/Experiment
Wang 2018	LM-RRT	Yes	No	2D	Yes	Indoor	Simulation/Experiment
Xinyu 2019	Bidirectional-RRT	Yes	No	2D	Yes	Indoor	Simulation/Experiments
Bohlin 2000	Lazy PRM	Yes	No	2D	Yes	Indoor	Simulation/Experiment
Karaman 2011	PRM* & RRT*	Yes	No	2D	Yes	-	Simulation
Ravankar 2019	ITC	Yes	No	2D/3D	Yes	Indoor	Simulation/Experiment
Lamini 2018	GA	Yes	No	2D	No	-	Simulation
Hu 2004	GA	Yes	Yes	2D	No	-	Simulation
Karami 2015	GA	Yes	No	2D	No	-	Simulation
Huang 2011	GA-PSO	Yes	No	2D	No	-	Simulation
Bi 2008	GA-FL	Yes	Yes	2D	Yes	-	Simulation
Ali 2019	LS/Sensor fusion	Yes	No	2D	Yes	Indoor/ Outdoor	Simulation/Experiment
Ali 2020	LS/FL	Yes	No	2D	Yes	Indoor/ Outdoor	Simulation/Experiment
Ali 2018	LS/Vision system	Yes	No	2D	Yes	Indoor/ Outdoor	Simulation/Experiment

**Table 2:** Shows distance comparison between GLS and other algorithms, measured in unit cells.

Maps	A				B				C			
Algorithm	PRM	RRT	A*	GLS	PRM	RRT	A*	GLS	PRM	RRT	A*	GLS
Trials												
1	807.71	797.21	697.77	663	694.53	709.94	611.14	579	593.91	580.85	477.97	463
2	782.16	881.74	697.77	651	697.57	713.55	611.14	583	676.37	537.18	477.97	411
3	800.85	802.04	697.77	692	671.06	789.11	611.14	501	590.54	506.87	477.9	392
4	702.00	719.00	697.77	678	669.37	675.72	611.14	536	610.73	563.23	477.9	397
5	789.55	889.44	697.77	667	746.00	787.44	611.14	589	492.79	460.74	477.97	467
6	854.25	839.82	697.77	698	783.12	698.78	611.14	452	574.80	531.30	477.9	398
7	694.29	830.40	697.77	587	754.60	600.35	611.14	632	741.34	529.27	477.97	387
8	777.30	748.67	697.77	588	758.82	777.92	611.14	547	617.53	521.96	477.97	388
9	843.70	737.65	697.77	465	724.56	612.40	611.14	548	460.26	495.11	477.97	365
10	767.20	800.23	697.77	697	694.35	662.49	611.14	519	692.04	790.11	477.97	397
11	767.23	774.09	697.77	411	613.80	638.74	611.14	553	799.70	706.27	477.97	214
12	738.25	787.16	697.77	610	719.50	623.65	611.14	530	745.91	537.29	477.97	310
13	794.71	877.68	697.77	627	783.36	661.09	611.14	626	644.82	513.38	477.97	427
14	721.37	870.13	697.77	529	686.14	794.71	611.14	592	505.29	565.83	477.97	329
15	772.06	714.39	697.77	707	743.69	693.78	611.14	524	548.92	637.76	477.97	437

**Table 3:** Shows searching time comparison between GLS and other algorithms, measured in seconds (s).

Maps	A				B				C			
Algorithm	PRM	RRT	A*	GLS	PRM	RRT	A*	GLS	PRM	RRT	A*	GLS
Trials												
1	2.22	4.48	137.37	1.33	4.39	4.84	69.84	1.06	4.48	3.50	54.84	2.98
2	3.77	7.77	137.51	2.58	4.14	3.16	68.81	1.21	3.06	4.58	55.24	2.11
3	3.65	5.52	140.06	2.30	3.91	3.79	69.56	3.50	4.68	5.45	56.11	3.75
4	2.62	4.50	135.70	2.43	3.60	3.25	68.61	2.56	3.36	3.20	54.46	2.90
5	3.01	10.35	144.00	2.49	3.88	3.11	67.83	2.73	3.17	7.98	53.97	3.14
6	3.61	11.28	136.28	2.70	4.15	2.69	70.72	2.99	3.83	6.13	55.21	2.83
7	2.72	4.98	138.71	1.73	3.55	2.74	69.16	2.70	3.33	3.20	52.90	2.25
8	4.46	10.38	134.80	2.41	4.08	4.05	68.36	3.48	4.50	2.94	54.21	2.59
9	3.97	7.64	134.68	2.61	2.84	3.77	69.60	1.35	3.36	9.48	54.27	3.10
10	3.01	6.49	139.57	1.24	3.40	2.97	69.89	2.40	4.76	3.66	53.54	3.44
11	2.94	5.11	134.93	1.72	3.18	4.17	67.70	1.65	3.37	2.45	54.67	2.13
12	2.71	8.01	137.43	2.43	3.92	3.90	69.68	2.16	2.92	4.48	54.13	2.16
13	2.99	5.77	136.05	1.14	3.25	5.23	72.66	2.40	4.06	2.57	53.02	2.17
14	2.77	3.61	136.90	1.14	3.63	5.06	69.48	2.14	3.22	10.85	57.02	3.09
15	3.44	4.51	138.14	1.69	3.99	3.15	70.90	1.59	4.14	4.34	54.65	3.08

**Table 4:** Shows comparison between GLS and other algorithms.

Environments	Simulation/ Experiments	Algorithm	Path Cost (mm)	Search Time (ms)	Path Smoothness (Low: When All Path Has Zigzag, Medium: When Zigzag Existed Partially In Path, High: Small or Non-zigzag Path)
Environment A Figure 11	Simulation	A*	478.34	27.89	Medium
		PRM	472.34	9.93	High
		RRT	506.05	3.50	Low
		GLS	493.75	3.59	High
Environment B Figure 11	Simulation	A*	540.15	84.47	Medium
		PRM	531.84	5.99	High
		RRT	639.25	8.08	Low
		GLS	579.25	4.17	High
Environment C Figure 11	Simulation	A*	516.14	24.88	Medium
		PRM	522.22	7.49	High
		RRT	30.05	3.64	Low
		GLS	662.58	2.33	High
Environment A Figure 15	Real-time Experiments	LS	244.52	6.37	Low
		GLS	143.29	1.51	High
Environment B Figure 15	Real-time Experiments	LS	300.29	2.89	Low
		GLS	118.88	0.26	High
Environment C Figure 15	Real-time Experiments	LS	336.83	5.94	Low
		GLS	205.12	0.34	High
